# The sequence structure and phylogenetic analysis by complete mitochondrial genome of kohlrabi (*Brassica oleracea* var. *gongylodes* L.)

**DOI:** 10.1080/23802359.2021.1966341

**Published:** 2021-08-18

**Authors:** Dengkui Shao, Yidong Ma, Xiaojuan Li, Sang Ga, Yanjing Ren

**Affiliations:** a Qinghai University (Qinghai Academy of Agriculture and Forestry Sciences), Xining, PR China; b Qinghai Key Laboratory of Vegetable Genetics and Physiology, Xining, PR China; c Comprehensive Agriculture and Animal Husbandry Service Center of Yushu Tibetan Autonomous Prefecture, Yushu, PR China

**Keywords:** Kohlrabi, mitochondrial genome, genome structure, phylogenetic analysis

## Abstract

Kohlrabi (*Brassica oleracea* var. *gongylodes* L.) is an important dietary rhizome vegetable in the Brassicaceae family. However, to date, few mitochondrial genomic resources have been reported for kohlrabi. In this study, we obtained the complete mitochondrial DNA sequence of 219,964 bp from an individual green kohlrabi. A total of 61 genes were annotated, including 33 protein-coding genes, 23 transfer RNA genes, three ribosomal RNA genes, and two pseudo genes. In addition, 1,001 open reading frames and five RNA editing sites were annotated. Relative synonymous codon usage analysis revealed significant difference in usage frequency of synonymous codon. Phylogenetic inference showed that kohlrabi is closely related to *B. oleracea* var. *botrytis.* This study provides a good foundation for further understanding the relationship and evolutionary origins among Brassicaceae crops.

Kohlrabi (*Brassica oleracea* var. *gongylodes* L. 2*n* = 2X = 18) is an important dietary rhizome vegetable in the Brassicaceae family, with its round swollen stem as the commodity organ (Zhang et al. 2015). It is widely cultivated in Europe, America, Canada, and Asia (Lim 2015). As a vegetable crop, kohlrabi possesses rich nutritional value for a healthy human diet, providing a high level of antioxidants and health-promoting substances (Rahim et al. 2017), such as vitamin C, vitamin E, carotenoids, ascorbic acid, and tocopherols (Jahangir et al. 2009; Park et al. 2012; Zhang et al. 2015).

Mitochondria are semi-autonomous organelles possessing their own genomes, and are responsible for plant energy production, metabolism, and cell homeostasis. Consequently, they are also known as the ‘power houses’ of cells. In addition, mitochondria play a vital role in development, reproduction, and various biochemical processes in plants by supplying ATP via oxidative phosphorylation (Palmer and Herbon 1988; Sloan et al. 2012; Liao et al. 2018). The length of plant mitochondrial (mt) DNA varies greatly—from 208 kb to 11.3 Mb (Sloan et al. 2012; Shen et al. 2019)—and often contains a large number of non-coding sequences, including gene spaces, repeat sequences, and introns (Wang et al. 2019). Many mt genome sequences of *Brassica* crops have been reported (Palmer and Herbon 1987; Yang et al. 2016; Hatono et al. 2017; Yang et al. 2018; Wu et al. 2019).

Fresh young and healthy leaves of kohlrabi were obtained from an individual green kohlrabi seedling planted in the field at Qinghai University (N36°42′; E 101°45′), Xining, China. Total genomic DNA of turnip was extracted from fresh and healthy leaves using the modified CTAB method (Porebski et al. 1997) and stored at Qinghai university. DNA purification and library construction were performed by the Shaanxi Breeding Biotechnologies Co., Ltd, according to the manufacturer’s instructions (Han et al. 2020) and sequenced using the Illumina HiSeq2500 platform. A total of 174,736,000 reads were obtained and filtered using fastp software (https://github.com/OpenGene/fastp), resulting in 5.24 Gb of clean data with a Q30 value of 91.19%. The draft kohlrabi mt genome was assembled using a complete mt genome of *Brassica oleracea* var. *capitata* (KU831325.1) as a reference, and further corrected by PE read mapping. The complete mt genome was visualized using DOGMA (Wyman et al. 2004). Genes in the complete mt genome were annotated using blast v2.6, tRNAscanSE (Chan and Lowe 2019), Open Reading Frame Finder (http://www.ncbi.nlm.nih.gov/gorf/gorf.html), and online software (http://www.prepact.de/prepact-main.php). A total of 61 genes were annotated, including 33 protein-coding genes, 23 transfer RNA genes, three ribosomal RNA genes, and two pseudo genes. In addition, 1,001 open reading frames and five RNA editing sites were annotated.

Relative synonymous codon usage analysis showed significant differences in usage frequency of synonymous codon. Frequency of UAA usage was the highest when encoding a termination codon. Frequency of GCU usage was the the highest when encoding alanine. The results showed that the synonymous codon encoding the same amino acid has a great preference choices in its usage. Scattered repeats analysis identified 245 repeats, including 128 forward repeats and 117 palindromic repeats. The phylogenetic relationships among 13 species and kohlrabi based on coding sequences was determined using RAxML v8.2.10（https://cme.h-its.org/exelixis/software.html） with the maximum-likelihood method and 1000 bootstrap replicates. *Oryza sativa* (NC 031333.1) was used as the outgroup. The results showed that kohlrabi (MW900252) is more closely related to *B. oleracea* var. *botrytis* (KJ820683.1) than to var. *capitata* (KU831325.1) (Figure 1), indicating similar evolution between kohlrabi and cauliflower. This study lays a good foundation for further understanding the relationships and evolutionary origins among Brassicaceae crops.

## Data Availability

The data that support the findings of this study are openly available in NCBI at (https://www.ncbi.nlm.nih.gov/nuccore/MW900252) under the accession no. MW900252. The associated BioProject, SRA, and Bio-Sample numbers are PRJNA721268, SRR14211765 and SAMN18713453. Phylogenetic analysis of fourteen species based on the mitochondrial genome CDS sequences. The mitochondrial sequence of *Oryza sativa* was used as the outgroup.
